# Royal Orthopædic Hospital

**Published:** 1902-04-19

**Authors:** 


### ROYAL ORTHOPÆDIC HOSPITAL.

### THE QUESTION OF REMOVAL.

No lack of interest could have been complained of on Thursday, April 10th, at the annual court of the Governors of the Royal Orthopædic Hospital, Hanover Square. By four o'clock the board room was full, and late-comers had to be content with standing-room.

At the table were Mr. Harry H. Marks, J.P., chairman; Mr. Ernest Flower, M.P., hon. secretary and deputy-chairman; Mr. Richard Biddulph Martin, M.P., J.P., hon. treasurer; Mr. H. A. Reeves, and other members of the committee.

It soon became evident that something of unusual interest was expected, and as the question of removal has been for some years under consideration, it was natural to conclude that a division of opinion existed among the governors. A small compact body, chiefly composed of ladies, seemed, judging by their votes, to be in opposition to the majority, though they sometimes refrained from voting altogether.

### The Minutes.

The meeting was the annual general court of governors, followed by a special court at which the governors were asked to sanction certain alterations in the laws, to ratify an agreement made by the committee of management for the disposal of the existing site, and to empower the committee to take the necessary steps for the acquisition of a new site and the erection of a new hospital thereon. The opposition, however, did not wait for the special meeting; it began at once, directly after the secretary had read the minutes of the last meeting.

Mr. Reeves wanted to know why the resignation of the hon. solicitor (Mr. Barnard Parker) had not been mentioned. Dr. Walsh said the proceedings were marked with negligence at the very outset, at which a lady in the corner said "Hear, hear." Mr. Reeves further wanted to hear at once about the grant from King Edward's Fund, which, however, he called the Prince of Wales's Fund, but on being requested to resume his seat he did so, and the minutes were passed.

### The Passing of the Report.

The next business was the annual report. The Chairman thought there was no controversial matter in it, unless it were the reference to the site, and as this would be discussed by the special court in a few minutes, he trusted it would not be entered upon now. The motion was seconded. Dr. Walsh suggested that the whole of the report should be passed, with the exception of that portion dealing with the site. He was prepared, if the Chairman and the meeting approved to discuss the site now. The Chairman said he was not prepared to agree to that.

Mr. Reeves asked why there was no reference in the report to the resignation of Dr. Greenlees, and why Mr. G. D. Hay and Mr. Cuerton had been elected, the one to the committee, and the other as vice-president. The Chairman declined to reply; he said it did not come into the report. It was at this juncture that Mr. Reeves accused the Chairman of wanting to muzzle the meeting. The Chairman said it was open to anyone to discuss the report, but not to ask any question which did not arise out of it, or which did not, in his judgment, do so. If Mr. Reeves wished to make a resolution regarding the omissions, he must do so on paper; he called Mr. Reeves to "Order, order." After some minutes Mr. Reeves handed in his resolution, "That the report be not accepted until the paragraph has been added dealing with the appointments and resignations."

This having been seconded, Dr. Walsh said it was natural, from what he heard, that some allusion should be required. The very fact of the omission pointed to the conclusion that it was done for some reason. He represented one of the most important medical papers, as well as being a governor, and the omission appeared to him to be out of order. Petty recriminations were not in his programme, but this omission aroused his feelings. "We don't seem," he added, "to be met with that spirit which should characterise a great charity."

The Chairman said that considering the fact that the resolution was seconded by a governor who became a governor only yesterday, and was not in order, it must be obvious that this court of governors had no power to amend a report made by the committee of management. They might accept it or reject it, but they must not tamper with it.

Mr. Reeves moved that the report be not accepted, and Dr. Walsh asked whether the Chairman's ruling was that the site might now be discussed.

The Chairman: I cannot rule on a point of opinion, only on a point of procedure. It would be a gratuitous waste of time to discuss it now, and again in a few minutes' time. It is perfectly open to Mr. Reeves to discuss the report. The question is that the report and accounts be accepted, on which Mr. Reeves has moved that they be not. The Chairman put the motion, and a number of hands went up.

Mr. Reeves said: "You needn't trouble to count."

The motion was declared carried, and Mr. Reeves, trying to address the meeting, was prevented.

### Further Objections.

The election of the committee was the next business. Mr. Harry Marks was elected, four ladies giving their votes against him; Mr. Ernest Flower and Mr. G. D. Hay came next. The former was elected; as to the latter, Mr. Reeves entered upon an elaborate explanation of why he was not eligible, with copious references to the rules, in which it is provided that only governors of twelve months' standing shall serve on the committee. He was again supported by Dr. Walsh, who demanded what Mr. Hay had done to merit the distinction. As long as he had an opportunity of protesting in public, he would not be a party to this overriding of the rules. Why was not Mr. Hay's name on the list?

The Chairman replied. He said the wish for accuracy and efficiency should be regarded. The reply might cause some dismay. The fact was that the report dealt with the history of the year ending December 31st; Mr. Hay was elected on December 27th, but he was not qualified to serve till about January 3rd. Mr. Cuerton was in the same case.

The election proceeded. Mr. James Head, M.P., Major F. I. Ricarde-Seaver, Mr. H. A. Reeves, Mr. Charles Read, and Mr. W. Gordon Campbell were re-elected, when Dr. Walsh cried out, "Oh, but we shall be swamped! These are all company promoters!"

The Chairman: Kindly resume your seat and behave like a gentleman. You said you came here in the public interest.

Dr. Walsh: I don't object to Mr. Gordon Campbell.

The Chairman: I call upon Dr. Walsh to withdraw his statement, which is in direct opposition to truth. "The affairs of this hospital," said Dr. Walsh, "have been for some years in the hands of gentlemen from the City, and they have not been conducted with what we consider success. We believe the hospital is being hurried into ruin. That is why I said there were more company directors coming on." He, however, withdrew his statement utterly. Mr. Frank Matthews was re-elected, and Mr. Athon Thorne was elected without difficulty, but the nomination of Mr. Thomas Clifford for re-election drew forth a statement from Mr. Flower; that gentleman's attendances, he said, had been very infrequent; he thought that if the committee found that he could give more time, it should be left with them to add his name. Mr. Reeves said the question had occurred before, and the Chairman had behaved in a most shabby manner. Mr. Clifford had then offered to resign, and his offer had not been accepted. Would the secretary read some of the other attendances? Mr. Flower said this was not necessary, as they had been already elected. Mr. Marcus Cuerton was next elected as a vice-president. He has given £50 to the hospital, and a portrait of the late Mr. Lonsdale, a former surgeon. Mr. Richard Biddulph Martin was re-elected hon. treasurer, and Messrs. Clark and Sully as hon. auditors.

On Mr. Charles Read being proposed for re-election as hon. surgeon, with Mr. H. A. Read and Mr. H. F. Baker, Mr. Reeves objected that Mr. Read had sent in his resignation three months ago. He read a letter to that effect. The Chairman said the proper course was to re-elect him, and leave it for the committee to deal with his resignation if it became necessary, as he feared it must. Mr. Read's health was very delicate.

### The Special Meeting.

This concluded the general meeting, and the secretary read the minutes of the last special meeting.

Mr. Head proposed that Law 13 be altered so as to admit the honorary officers to sit on the committee as *ex officio* members, with full voting powers. Mr. Flower seconded, and Mr. Reeves objected to Mr. Biddulph Martin's advent on the committee. "Did you express a wish to be on it?" he inquired, addressing that gentleman. Mr. Biddulph Martin said he did not. And what on earth did they want with their architect? added Mr. Reeves.

The Chairman said it was in view of the buildings, which would extend considerably above the earth. The hon. officers, he added, were not there to be cross-examined. The motion was carried. The next law dealt with the election of the hon. surgeons, placing it more fully in the hands of the committee of management. Mr. Reeves held that this was a medical matter; what, he asked, would happen if the medical staff did not concur in the choice of the committee? The Chairman said he thought it was for the committee to judge of the eligibility of a surgeon, after consultation with the medical staff. In the event of the medical staff not agreeing, the committee would take it upon themselves to appoint. He was not prepared to accept Mr. Reeves' amendment. The motion was carried.

### The Site.

The Chairman next moved what he described as the resolution of the day, "That the agreement made by the committee of management for the disposal of the existing site be ratified, and that the governors authorise the committee to take all necessary steps for the acquisition of a new site and the erection of a new hospital." The question of rebuilding, he said, had been before the committee for six years, and the matter had now become urgent, and, in the opinion of all interested in the hospital's welfare, removal was inevitable. The work had been excessively hampered and frequently interrupted, owing to the unsitableness of the site. Wards had frequently been closed owing to outbreaks of scarlet fever. The institution was closed entirely last year for reconstruction of drainage, and altogether over £2,160 had been spent on repairs, an average of £432 since 1897. This was necessary to keep the hospital open at all. The amount would have maintained the 10 beds which they had been obliged to close. The County Council had served notices, and some part of the alterations had been carried out to meet their requirements. In 1899 the committee then in office were prepared to accept £28,000 for the whole of the site; upon the present committee coming in, the guidance of Sir John Whittaker Ellis and his firm was sought, and in their report of January, 1900, they placed the value at £36,000. After further consultation, and advice from two other firms, an offer of purchase was received for £37,700, and in May last year the committee obtained the sanction of the Charity Commissioners and of the trustees of this charity to accept that offer. The negotiations, however, fell through on a matter of detail, and in the latter part of the year negotiations were opened with another purchaser. This offer the trustees considered favourable, and he would recommend its acceptance. It had been sanctioned by the Charity Commissioners, and the committee invited the approval of the governors. The only opposition came from one or two gentlemen who were desirous of rebuilding on the present site. The matter had been discussed with them from every possible and, he might say, impossible point of view. The committee were satisfied that the present long narrow plot was not at all suitable for the purpose, and indeed in all respects was essentially unsuitable for a hospital on modern lines. This was also the opinion arrived at by the late committee. It was also the opinion of King Edward's Hospital Fund, which made a special grant on the condition that it should be allocated to the removal of the site.

### A Piece of Arithmetic.

The speaker then went into financial details. The ground rent offered was £1,400, with the option of purchase at £40,000. Another site was obtainable at £500 ground rent, and it was on the basis of selling the old site and acquiring a new one that his figures were founded. There was an awkward hiatus between the terms of the two leases, which would not run concurrently; but he pointed out that they were not bound, by accepting the offer referred to, to take the proffered site. The ideal lease would be for 99 years, the two running side by side. But he would assume that the lessees decided to buy, at £40,000. The cost of erecting and furnishing a new building on modern lines was estimated at £25,000. This would leave a balance of £15,000 to be invested. A sinking fund must also be provided by an annual payment of £60. Interest at 3¾ per cent. on the investments would produce within £62 10s. of the total cost of the new hospital. The remainder would have to be found out of the ordinary resources of the hospital, and this £62 10s. would be the net cost to the institution. At the end of the term the £25,000 spent on building would come back, and the whole £15,000 would be intact.

At this point there were interruptions, but the Chairman asked to be allowed to finish his arithmetic. It was not certain that the committee would sell. Assuming that the option of doing so were not exercised, and that the lessee continued to pay £1,400 ground rent, the amount for the new buildings would then have to be borrowed, and he proposed to borrow at 3¾ per cent.

Mr. Matthews remarked "That would come to the same thing."

The Chairman: Let us finish our sum. There are several points not yet taken into account. There is an average expenditure of £432 on repairs. Then I have not allowed for any funds being raised by appeals, or for an enhanced subscription list, which it may be anticipated will accrue as a result of the public notice we shall bring upon ourselves by removing the hospital from an insanitary site.

### Opposition.

The resolution was seconded by Mr. Ricarde-Seaver, and opposed by Dr. Walsh, who characterised the methods of the committee, in their dealings with financial matters, as almost amounting to criminal recklessness. The late committee, he said, decided to sell for £28,000, and on that occasion he was himself greatly impressed by the charm of Mr. Harry Marks' manner, though at the same time convinced that a wrong was being done. He said he had shorthand notes of the meeting he referred to, but when the Chairman challenged him to produce them he acknowledged that he had not brought them with him.

The Chairman: Try again, Dr. Walsh; you were badly instructed. After consulting with Mr. H. A. Reeves, Dr. Walsh said he was quite right about the facts, but had had the wrong date in his mind. He acknowledged that on another occasion Mr. Marks opposed the sale.

The Chairman again challenged Dr. Walsh to produce one shred of evidence, and Dr. Walsh appealed to Mr. Barnard Parker. The Chairman said he had the greatest respect for that gentleman, because he never talked about a subject he did not understand.

Dr. Walsh said the late committee were thrown out because they wanted to sell, and that the new committee were elected on the opposite policy. At this there were cries of "No, no," and "Quite right," the latter chiefly from Mr. Reeves. One gentleman explained that there was no pledge whatever as to the policy of the new committee.

Dr. Walsh said of course he had great difficulty in getting at the truth of the matter. He would change his ground. He would say this: that the reasons against the sale were very strong indeed. If they sold, they would be selling their birthright. They were going to spend the purchase-money on bricks and mortar. Why should they deal with a hospital as they would not with private property? Why should they pay rent when the annual value of their own property was increasing? The value was £28,000, and it had gone up.

That, he was told, was not the value of the site. He continued: The old committee wanted to sell it, and that was why they were turned out. "We," said Dr. Walsh, "sent up the value to £36,000. We have only to stick here, and it will go up still higher." And at last he came to his alternative scheme. He proposed to build shops at the Oxford Street end of the site. That scheme, he said, had never been discussed. It might be called unbusinesslike, but its promoters were prepared to fight for the hospital and the medical charities, which were the only things they cared for. Whether they voted to-day or not, they had not done with the matter. If they built shops, the value of the site would go up to £63,000, and he claimed that the price had been sent up £12,000. Why not get a competent authority to advise, or why not put the site up to public auction in Tokenhouse Yard? He was talking to business men; he appealed to their business instincts. This was the first time there had been an explanation of the scheme detailed by Mr. Marks. The figures should have been laid before the governors before.

### A Parthian Shot.

He came now to his last point. This meeting was illegal. Some sensation was produced by this statement, and Dr. Walsh proceeded: "We have a charter. Kindly refer to page 3. You will read—'We (that is, Queen Victoria) direct that an annual Court of the Governors of the said hospital shall be held in February or March in every year.' Gentlemen, is this March or April?" At this there was much laughter, and Dr. Walsh proceeded to follow up his advantage. The consequences of this meeting, he said, might be great. Anyone who bought this hospital did so on an insecure tenure. Further, the trustees were responsible. He hoped and trusted that the governors would believe that he came there simply as a matter of public duty. He had endeavoured to keep himself under control, and he trusted that whatever decision was arrived at would be for the good of the Royal Orthopædic Hospital.

### In Support of the Scheme.

Mr. Ernest Flower said that whether the notoriety which the hospital would gain by this meeting would be a good thing or not was open to question. However pure Dr. Walsh's motives were, the committee at least claimed this, that they could not hope to obtain even the remuneration likely to accrue from copy supplied to a medical journal What they proposed was, in their opinion, for the best interests of the hospital, and if they had any doubts, these were dispelled by the Council of King Edward's Fund, and by the Charity Commissioners, who, of course, only gave their sanction on the financial merits of the scheme. Take a body like the Council of King Edward's Fund. They said the hospital was doing its very best with inadequate premises. They made a grant, and they coupled it with the statement that the site was inadequate, and that to stay was to inflict the expenditure of an unnecessary sum of money on repairs. Talk about independent reports, could there be a better independent report than that? This might be fairly said—that that body and the new committee had come to one opinion as to the necessity of leaving the premises. He submitted that the one point on which they must concentrate their attention was whether this scheme did or did not provide for their getting the best possible terms for the disposal of their property. They had the opinion of the Charity Commissioners, who had sent their own independent surveyor; and although the committee had been criticised as financiers, he was not himself a director of any company (nor was he aware that there was anything very wrong about such a position), but he supposed he was tarred with the same brush as his colleagues. After all, directors of companies were business men, and it was just the possession of that business acumen which induced the shareholders to place them in such a responsible position. Was there now enough evidence for the scheme? In his opinion it was overwhelming; if the value of the site might go up, it might also come down, and having had medical advice as to the necessity of removing, he sincerely hoped the governors would concur in the decision of the committee, who were acting solely in the interests of the hospital.

Mr. Richard Biddulph Martin supported the scheme. Personally, he would like to see the hospital moved to the country, where the children could have the benefits of fresh air. He had been over the scheme, and had visited several hospitals on behalf of King Edward's Fund, in company with one of the most eminent physicians of the day, and he did not see how it was possible to get the accommodation for the staff in the limits of this site. At first he felt that unless the hospital could be moved to the country he would leave it in Hanover Square, but on going carefully into the whole question, he could see no alternative to a removal. The site was as ill-adapted as it could possibly be, and wanting in everything that a hospital ought to have. It could not be patched up. Even with the shops at the bottom he did not see how they could build so as to get the required accommodation. His opinion was entirely in accordance with that of the Chairman. It was impossible to have what they would like, and they must put up with what they could get. The motion put from the chair was the wisest they could have, and it merited their serious consideration.

### Mr. Reeves Again.

Mr. H. A. Reeves, who had been quiescent for a long time, got up at this point and said that the man he wanted to support him had not come. It might be the last time he himself would address the governors, and he wished to give his opinion of the Council of King Edward's Fund. They were a parcel of amateurs; what did they know about medical matters? For fifty years there had not been a death in the hospital, and now they said it was insanitary. They said it was old, and that it was "desirable to move." He had never heard of such a feeble resolution as that supported by Mr. Biddulph Martin. When the Chairman said more accommodation was wanted, he thought it was for the patients. He found it was for the staff! What did they want with more accommodation than they had? The very plans of the scheme were enough to condemn the site in Great Coram Street. The hospital would be confronted by tall workmen's dwellings, and infection would be wafted into it. . . .

The Chairman interrupted. No particular site, he said, was under discussion.

"Have you, then," asked Mr. Reeves in intense scorn, "no site? Are you going to sell this hospital, and have you no place for the patients to go to?" The Chairman was silent. Mr. Reeves further declared that King Edward's Fund had absolutely withdrawn its demand for removal; that it had given £200 last year, after withholding its donation in 1900. Mr. Flower explained that the grant was given with a strong expression of opinion from the Council, who desired that the £250 should be set apart for the building; it was given on the understanding that the committee intended to move. He read a letter from Sir Savile Crossley in support of this. Mr. Reeves demanded the date, and was told, January, 1899. He objected that this was 1902, and was further told that the same stipulation had accompanied the grant of £200 in 1901.

It was at this point that Mr. G. D. Hay intervened, and objected to "a lot of erratic talk of no value."

Mr. Reeves said it was stated in the *Times* that the acceptance of the grant got the hospital into trouble. The Chairman attempted to answer, and Mr. Reeves said, "Mr. Chairman, will you kindly be quiet?" and went on with his remarks on the alternative scheme propounded by Dr. Walsh. Then Mr. Reeves charged Mr. Marks with having wished to sell his property, Loughton Hall, to the hospital for a site.

An Extraordinary Statement.

The Chairman denied the charge. He said it was a fabrication, a deliberate falsehood, and he challenged Mr. Reeves to bring forward any support whatever to bolster it up.

Mr. Reeves: I have come here to manufacture lies, have I? There were cries of "Time," and Mr. Reeves only said, "He meant well, no doubt."

Mr. Barnard Parker inquired, "What on earth has this got to do with it?"

Mr. Reeves said that Mr. Sydney Holland considered this a magnificent site and asked why they did not build there.

The Chairman: The property of Loughton Hall was sold in 1898 to the Duke of Norfolk and other Roman Catholics, and I was elected to this committee in 1899. That is enough to characterise the statement just made as a deliberate falsehood.

Mr. Flower moved that the question be now put, and the resolution was carried by a large majority. Mr. Reeves said something to the effect that further action would be taken, and the meeting dispersed, leaving Mr. Reeves alone in the Board-room.

Since this meeting the following letter has been forwarded to the *Times* by Messrs. Lewis and Lewis, Ely Place, Holborn, under date April 12th:—"In your issue of to-day appears a report of the annual meeting of the court of governors of this hospital, at which meeting Mr. H. A. Reeves, honorary surgeon to the hospital, made certain disparaging remarks upon Mr. H. H. Marks, the chairman of the hospital. As solicitors for Mr. Marks we called upon Mr. Reeves to retract such statements, and to offer him an apology, otherwise proceedings would be taken. Mr. Reeves' solicitors have to-day written as follows:—'He instructs us to say that he made no statement at the meeting yesterday which was in any sense intended to suggest or convey that Mr. H. H. Marks had been guilty of dishonourable or discreditable conduct as chairman of the hospital, and that if, in a moment of great excitement, he should unwittingly have been betrayed into saying anything to the meeting which was capable of being construed into any reflection upon the honour or character of your client, he most sincerely regrets having done so, and begs to apologise to Mr. Marks for any injury to his feelings that the incident has caused him.' In justice to Mr. Marks we shall feel obliged by your inserting this letter in your next issue."

